# Extra-abdominal and intra-abdominal *FET*::*CREM* fusion mesenchymal neoplasms: comparative clinicopathological study of 9 new cases further supporting a distinct potentially aggressive sarcoma and report of novel sites

**DOI:** 10.1007/s00428-024-03917-2

**Published:** 2024-09-09

**Authors:** Abbas Agaimy, Morgan Blakely, Gerben E. Breimer, Annett Hölsken, Sjors A. Koppes, Norbert Meidenbauer, Johannes A. Rijken, Arno Schad, Adrian G. Simon, Robert Stoehr, Justin A. Bishop, Nasir Ud Din

**Affiliations:** 1 Institute of Pathology, University Hospital Erlangen (UKER), Friedrich-Alexander University Erlangen-Nürnberg (FAU), Krankenhausstraße 8-10, 91054 Erlangen, Germany; 2 Comprehensive Cancer Center Erlangen-EMN (CCC ER-EMN), Erlangen, Germany; 3 Department of Pathology, Kaiser Santa Clara, Santa Clara, CA 95051 USA; 4 Department of Pathology, University Medical Center Utrecht, Utrecht, The Netherlands; 5 Department of Pathology, Erasmus University Medical Centre, Rotterdam, Netherlands; 6 Department of Internal Medicine 5-Hematology and Oncology, Erlangen University Hospital, Friedrich Alexander University of Erlangen-Nuremberg, Erlangen, Germany; 7 Department of Head and Neck Surgical Oncology, University Medical Center Utrecht, Utrecht, The Netherlands; 8 Department of Pathology, University of Mainz, Mainz, Germany; 9 Department of Pathology, University Hospital of Cologne, University of Cologne, Cologne, Germany; 10 Department of Pathology, University of Texas Southwestern Medical Center, Dallas, TX USA; 11 Department of Pathology and Laboratory Medicine, Aga Khan University Hospital, Karachi, Pakistan

**Keywords:** Next generation sequencing, Ewing sarcoma, Precision medicine, Genetic landscape, Profiling, Constitutional symptoms

## Abstract

With the wide use of RNA sequencing technologies, the family of *FET::CREB* fusion mesenchymal neoplasms has expanded rapidly to include potentially aggressive neoplasms, not fitting any well established WHO entity. Recently, a group of intra-abdominal *FET*(*EWSR1/FUS*)*::CREB*(*CREM/ATF1*) fused unclassified neoplasms has been reported followed by recent recognition of an analogous extra-abdominal category of unclassified neoplasms carrying *EWSR1::ATF1* fusions. We describe 9 additional tumors (5 extra-abdominal and 4 abdominal) carrying an *EWSR1::CREM* (*n* = 8) and *FUS::CREM* (*n* = 1) fusion. Patients were 7 females and 2 males aged 10 to 75 years (median, 34). Extra-abdominal tumors originated in the head and neck (2 sinonasal, 1 orbital) and soft tissues (1 gluteal, 1 inguinal). Abdominal tumors involved stomach (2), mesentery (1), and kidney (1). Tumor size ranged from 3.5 to 11 cm (median, 6). Treatment was radical surgery with (5) or without (2) neo/adjuvant radio/chemotherapy. Extended follow-up of 5 patients (21–52 months; median, 24) showed an aggressive course in two (40%); one died of disseminated metastases 52 months after several intensified chemotherapy regimens, and one was alive with progressive abdominal disease at 21 months. The immunophenotype of the two subcohorts was significantly overlapping with variable expression of EMA (7 of 8), keratin AE1/AE3 (5 of 9), CD99 (4 of 7), MUC4 (2 of 8), ALK (3 of 8), synaptophysin (3 of 9), chromogranin (1 of 8), CD34 (3 of 6), CD30 (1 of 6), PAX8 (1 of 7), and inhibin (1 of 7), but no reactivity with desmin (0 of 8), S100 (0 of 8), and SOX10 (0 of 8). This series further solidifies the notion that *FET::CREB* fusions are not limited to the triad of angiomatoid fibrous histiocytoma, clear cell sarcoma, and malignant gastrointestinal neuroectodermal tumor, but characterize an emerging family of potentially aggressive neoplasms occurring at both intra- and extra-abdominal sites. These tumors underscore the promiscuity of the *FET::CREB* fusions and highlight the pivotal role of phenotype-oriented classification of these neoplasms that share the same genotype, still featuring significant biological and behavioral distinctness.

## Introduction

The FET (***F****US/****E****WSR1/****T****AF15*) family is a group of structurally and functionally homologous genes mapped to different chromosomal regions and encoding for the FET proteins, a group of RNA-binding proteins [1]. These ubiquitously expressed proteins are involved in the processes of gene transcription, post-transcriptional modification, and regulation of gene expression, thereby contributing to regulation of vital cellular processes and DNA damage repair [1].

The CREB family is a group of DNA-binding transcription factors that bind to the cAMP DNA region [2, 3]. Cyclic adenosine monophosphate (cAMP) is an important intracellular messenger involved in intracellular signal transduction and hence influences diverse vital cell functions [2, 3]. The CREB family of transcription factors includes CREB1 (cAMP responsive element-binding protein-1), CREM (cAMP responsive element modulator), and ATF1 (activating transcription factor 1) [2, 3]. Genes encoding these three transcription factors possess a high degree of structural and functional homology and are involved in diverse processes of gene transcription regulation [2, 3].

Gene fusions involving members of the *FET* and *CREB* families have been increasingly recognized as recurrent oncogenic events, driving oncogenesis in anatomically, phenotypically, and genetically diverse mesenchymal neoplasms occurring in soft tissue, bone, and visceral sites [4]. Traditionally, *FET::CREB* fusions have been limited to a triad of distinctive soft tissue neoplastic entities defined by variable combination of clinicopathological, morphological, and immunophenotypical characteristics: clear cell sarcoma of soft tissue (CCS; predominantly *EWSR1::ATF1* fused [5]), angiomatoid fibrous histiocytoma (AFH; predominantly *EWSR1::CREB1* fused [6]), and malignant gastrointestinal neuroectodermal tumor (MGNET; predominantly *EWSR1::ATF1* fused [7]). However, this triad of *FET::CREB* fusion neoplasms became increasingly challenged by the recognition of morphologically distinct entities occurring at different anatomic sites with or without phenotypic analogy to the aforementioned well-defined triad of entities. Specifically, primary intrapulmonary myxoid sarcoma and intracranial myxoid sarcoma have been widely accepted, but with an as-of-yet controversial relationship to the myxoid variant of AFH [8, 9]. Additionally, a *FET::CREB* fused neoplasm with morphological and phenotypic analogy to MGNET but occurring at extraenteric sites has been reported [10].

Recently, a group of abdominal mesenchymal neoplasms driven by *FET::CREB* fusions, but not mirroring any of the above-defined entities became increasingly recognized with the majority harboring *EWSR1/FUS::CREM*, and a minority carrying an *EWSR1/FUS::ATF1* fusion [11–14]. Some of these abdominal tumors affected the female [13] and male [14] gonads and have been reported by diverse and potentially confusing terms. Finally, our group has recently described a series of aggressive extra-abdominal soft tissue and bone neoplasms showing significant morphological and phenotypic analogy to those intra-abdominal neoplasms and carrying *EWSR1::ATF1* fusions, otherwise not fitting any defined entity [15]. We herein describe 9 *FET::CREM* fusion neoplasms with a significantly overlapping morphology and immunophenotype occurring at extra-abdominal (*n* = 5) and intra-abdominal (*n* = 4) sites. This series should help clarify the nosology of the emerging family of unclassified *FET::CREB* neoplasms.

## Material and methods

Eight tumors were identified in the consultation files of the authors and one in the routine files of the Institute of Pathology, Erlangen, Germany. Included were only tumors that have been judged by expert soft tissue pathologists to lack features of the defined WHO-listed triad of *FET::CREB* fusion soft tissue entities (CCS, AFH, and MGNET), by morphology and immunophenotypic characterization only, without consideration of the genotype. Accordingly, all these tumors were considered unclassified by the current WHO soft tissue and bone tumor classification [16]. The tissue specimens were fixed in formalin and processed routinely for histopathology. Due to the consultation nature of the cases, immunohistochemistry (IHC) was performed in different laboratories and the stains applied varied from case to case, based on tissue availability and initial differential diagnostic considerations (details of the staining protocols and antibody sources are available upon request).

### Next generation sequencing

For Cases 1, 3, 6, 7, and 9, RNA was isolated from formalin-fixed paraffin embedded (FFPE) tissue sections and subjected to targeted RNA sequencing using the Illumina TruSight Oncology 500 RNA Panel (https://emea.illumina.com/products/by-type/clinical-research-products/trusight-oncology-500.html) as previously described [17]. For Case 4, RNA was isolated from FFPE tissue sections and subjected to RNA sequencing. Library preparation and sequencing were performed according to the protocols outlined by Hehir-Kwa et al. [18]. Case 5 Case underwent targeted RNA sequencing, as previously described [19]. Case 2 and 8 were analyzed using the Archer FusionPlex-Panel Sarcoma on NextSeq (Illumina) and MiSeq (Illumina) with Archer Analysis Software, respectively.

## Results

### Clinical features

The affected patients were 7 females and 2 males aged 10 to 75 years (median, 34) (Table 1). Extra-abdominal tumors originated in the head and neck (2 sinonasal, 1 orbital), and in the gluteal (1) and inguinal (1) soft tissues. Intraabdominal tumors involved the stomach (2), mesentery (1), and kidney (1). Radical surgery with (5) or without (2) neo/adjuvant radio/chemotherapy was the treatment. Extended follow-up was available for 5 patients (range, 21–52 months; median, 24). Two patients (40%) developed progressive disease. One patient with gluteal primary developed disseminated bilateral lung metastases at 12 months and cardiac and sacral region metastases at 24 months. He received multiple intensified chemotherapies over more than 4 years utilizing different aggressive regimens (see Table 1). He died of widely disseminated progressive metastases 52 months after primary diagnosis. Another patient with gastric primary who received neoadjuvant chemotherapy followed by multivisceral radical surgery developed progressive intra-abdominal metastases (omental, paragastric) after 1 year and was alive with progressive recurrent/ metastatic intra-abdominal disease at 21 months. Overall, one of three extra-abdominal and one of two intra-abdominal tumors either resulted in the death of the patient or was incurable and progressive at the last follow-up, indicating an overall progressive course in 40% of patients.

**Table 1:** Clinical features of extra-abdominal (1–5) and intra-abdominal (6–9) *FET::CREM* fusion neoplasms

No	Age/sex	Site	Size cm	Clinical symptoms	Treatment	Tumor regression	Regional nodes	MTS (interval)	Outcome
1	31/M	Pelvis/gluteal	NA	Constitutional symptoms during metastatic disease (high CRP, thrombocytosis)	R1 surgery + radiotherapy (66 Gy), then 2 × cycles adriamycin (3 × 20 mg/m^2^)/ifosfamid (2 × 3000 mg/m^2^) for lung MTS, then 3 × cycles (Ewing-Protokoll), 2 × cycles cyclophosphamide/topotecan, then pazopanib, then pembrolizumab, 5 × cycles irinotecan 25 mg/m^2^, temozolomid 50 mg/m^2^), then RIST-Protokoll	Mixed response**	Positive nodes in resection, PET + inguinal nodes but not verified	Disseminated lungs (12 mo) + heart (24 mo) + sacral	DOD (52 mo)
2	29/F	Orbita	NA	Exophthalmos	Partial resection, then 4 cycles doxorubicin/ifosfamid (“Ewing-Protokoll) + radiotherapy (60 Gy), then exenetratio orbitae (R0)	50%	Not sampled	No	NED (24 mo)
3	38/M	Inguinal area	NA	NA	NA	NA	NA	NA	NA
4	51/F	Nasal cavity	3.7	Unilateral nasal obstruction	Resection (+ re-resection without residual disease)	–	Not sampled cN0	No	Recent case
5	62/F	Nasal cavity	3.5	Persistent nasal obstruction	Resection then 6000 cGy adjuvant radiation	–	Level 2a neck node core biopsy negative for carcinoma	No	NED (22 mo)
6	34/F	Right kidney	7.5	Hematuria, sudden flank pain	Radical nephrectomy + 7 cycles of adjuvant chemotherapy*	–	Not sampled	No	NED (50 mo)
7	10/F	Gastroesophageal Junction	11	Hematemesis + constitutional symptoms	5 cycles chemotherapy (Ewing protocol) + radiotherapy, then partial gastrectomy + omentectomy + splenectomy + diaphragm resection	10%	0/9	Omentum, paragastric	AWD (progressive intra-abdominal) at 21 mo
8	75/F	Gastric wall	11	Vomiting, upper abdominal pain	Gastric wedge resection	–	0/1	No	NED (11 mo)
9	23/F	Mesentery (2 nodules)	6 + 4.2	Constitutional symptoms (fever, high CRP, fatigue, dyspnea, anemia, thrombocytosis), paraneoplastic encephalitis	Right hemicolectomy + omentectomy	–	2/16	Snychronous regional nodes	NED (4 mo)

^*^Details not available; **Mixed response of lung mets on imaging; *AWPD*, alive with progressive disease; *DOD*, died of disease; *NED*, no evidence of disease

### Pathological findings

**Fig. 1:**
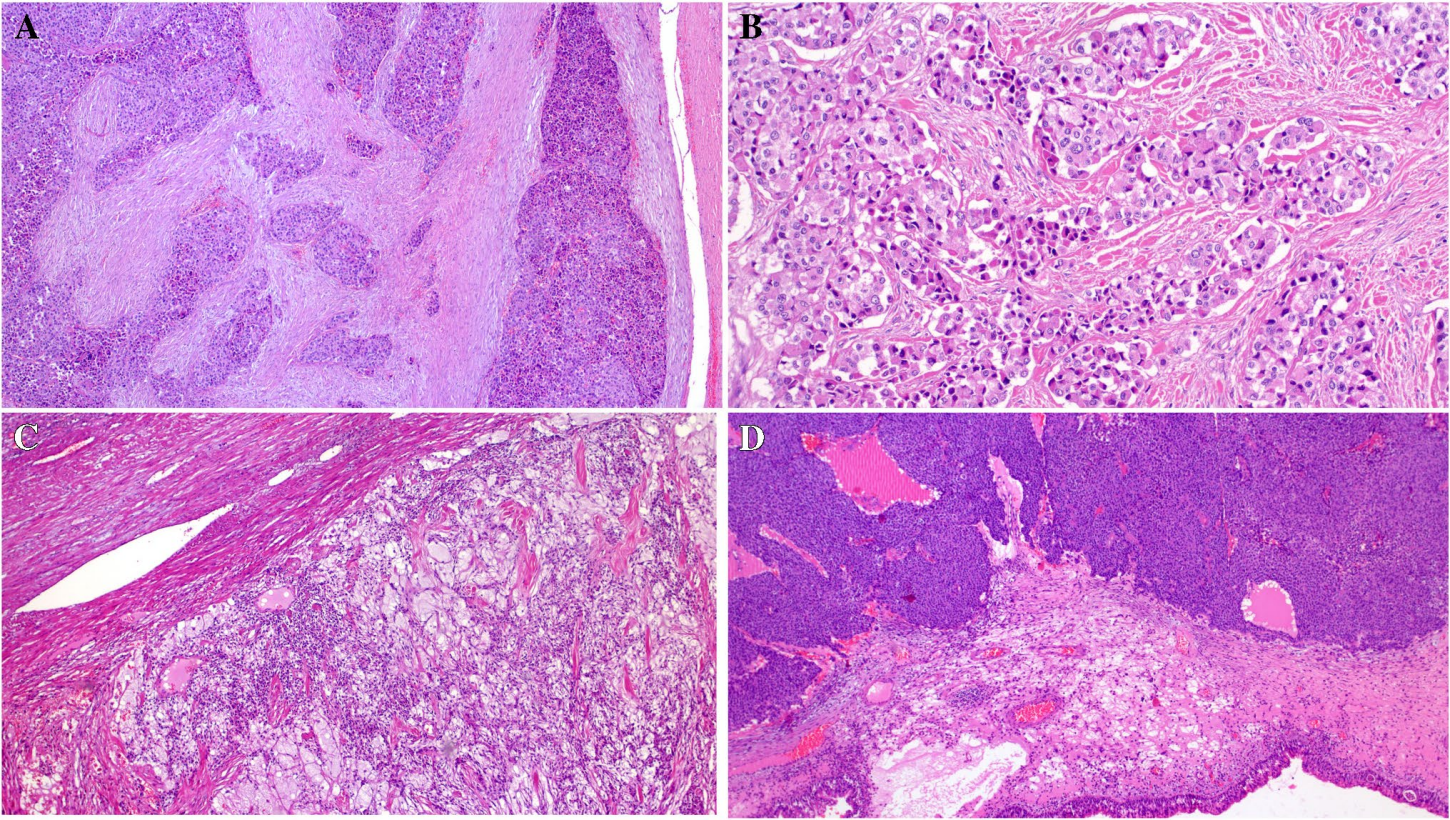
Low-power view of *FET::CREM* fusion neoplasms. **A** Extensive nesting and lobulation within prominent desmoplastic stroma. **B** Higher view of the same tumor showing smaller irregular clusters of monomorphic cells within the fibrous stroma. **C** This renal tumor was the only case with a fibrous pseudocapsule separating the tumor from the adjacent renal parenchymal (upper left). **D** Highly cellular sinonasal tumor showing irregular borders lacking lymphoid cuffs

**Fig. 2:**
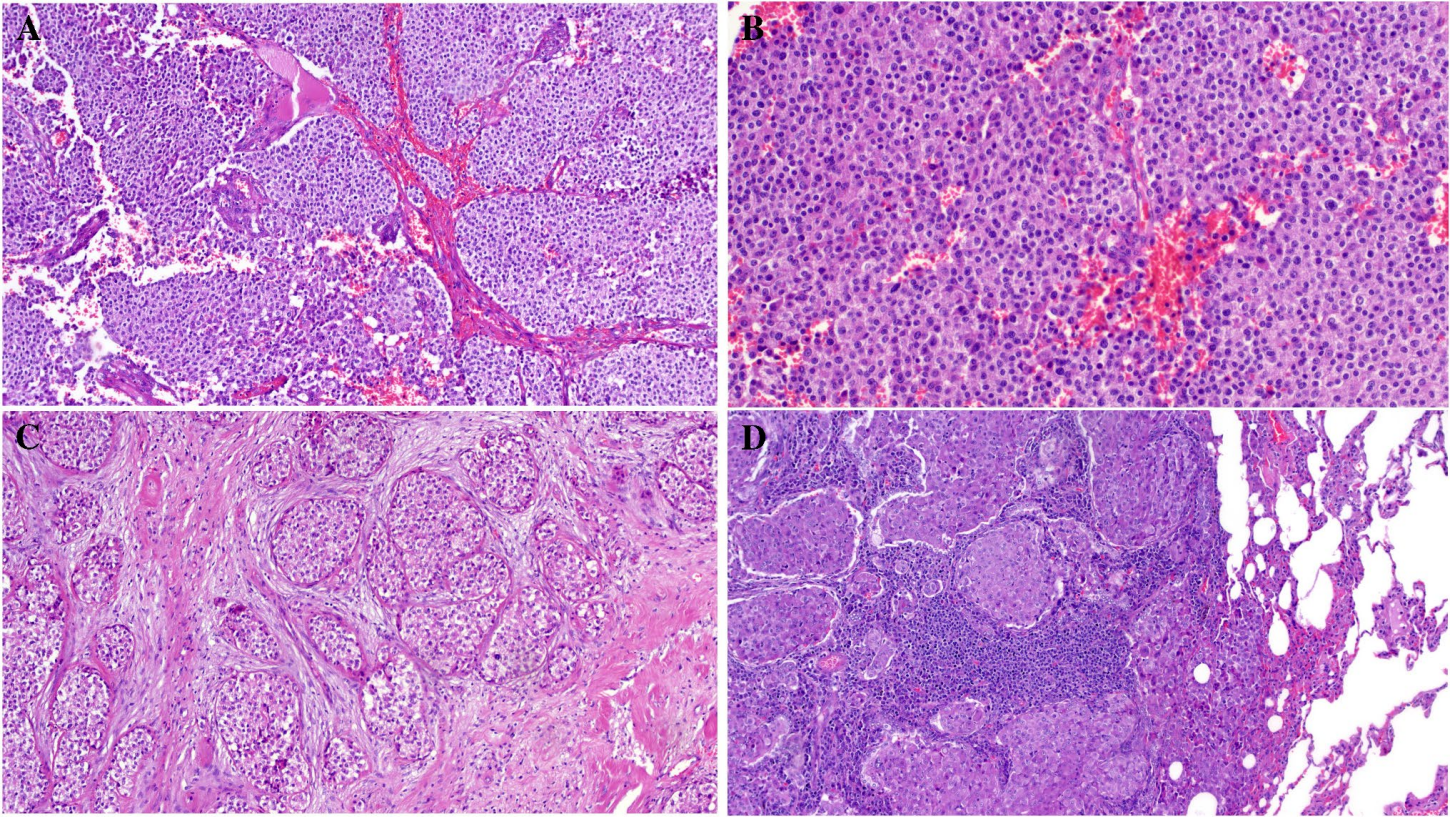
**A** Large solid organoid aggregates within vascularized stroma mimicking a neuroendocrine neoplasm. **B** Higher magnification of **A**. **C** Prominent “Zellballen”-like organization (together with synaptophysin expression) justified an original diagnosis of paraganglioma in this gluteal soft tissue mass. **D** Lung metastasis from the same tumor retained the organoid paraganglioma-like pattern

**Fig. 3:**
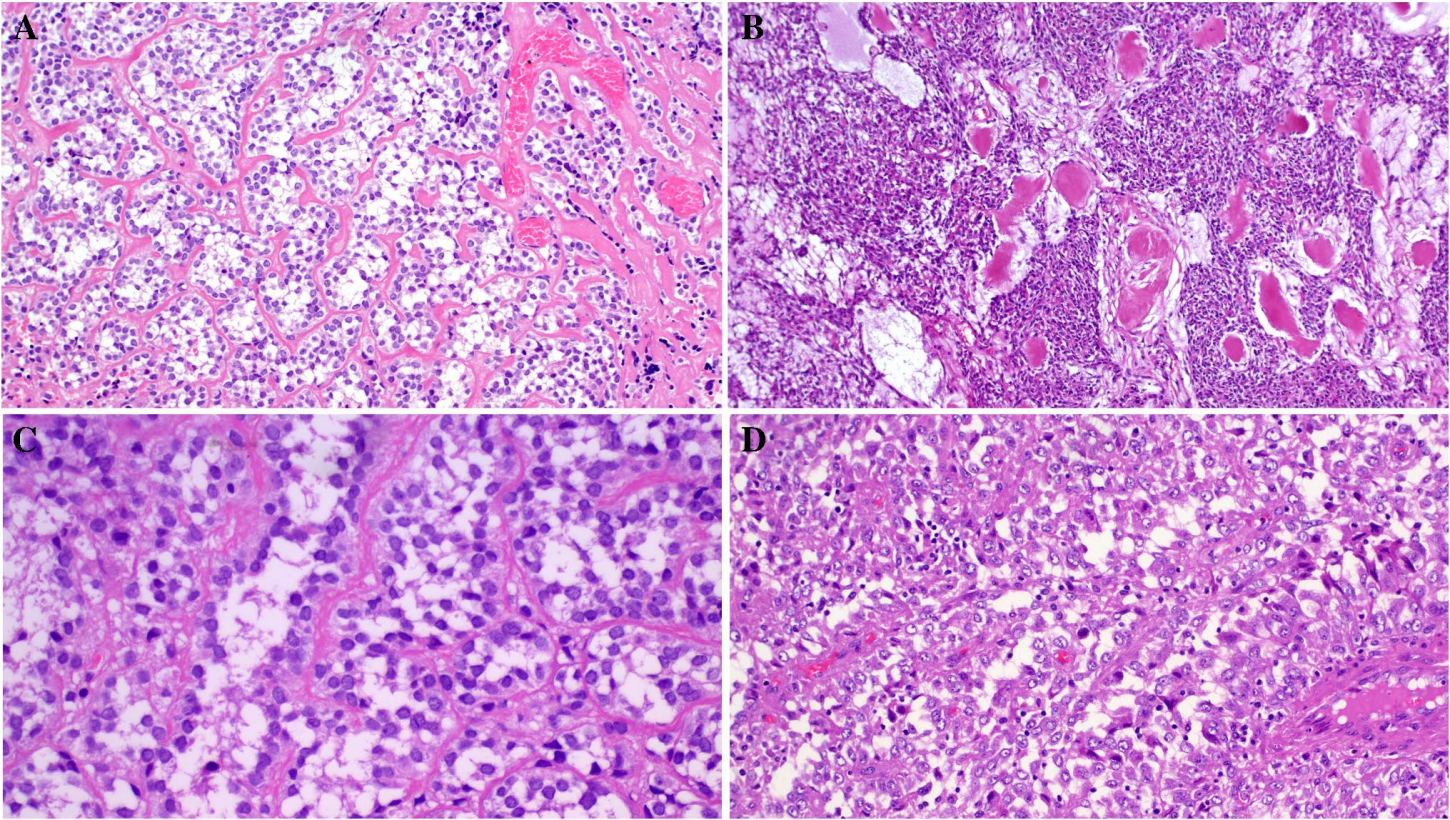
Stromal hyaline deposits either surrounding organoid tumor cell aggregates (**A**) or forming amianthoid-like structures (**B**) were seen in three tumors. Pseudoalveolar arrangement was noted in tumors with clear cells (**C**) or with pale-eosinophilic larger epithelioid cells (**D**)

**Fig. 4:**
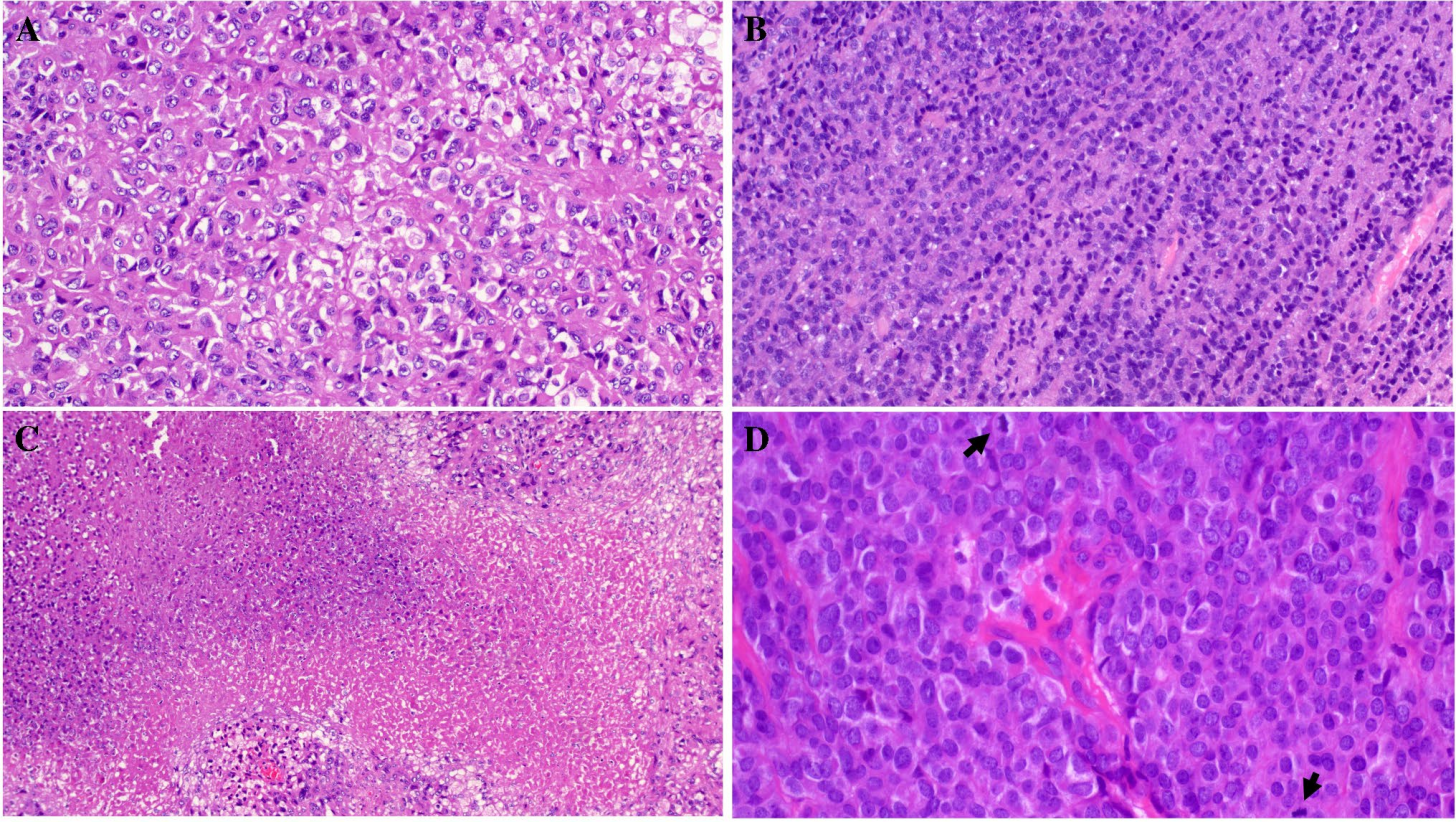
The cytology of the tumors ranged from large epithelioid cells with pale-eosinophilic to clear cytoplasm (**A**) to smaller dark stained round cells with scanty cytoplasm (**B**). Note the single cell file pattern in **B**. **C** Extensive geographic necrosis was seen in this abdominal tumor. **D** Brisk mitotic activity (arrows) in a sinonasal tumor, note non-descript round cell morphology

**Fig. 5:**
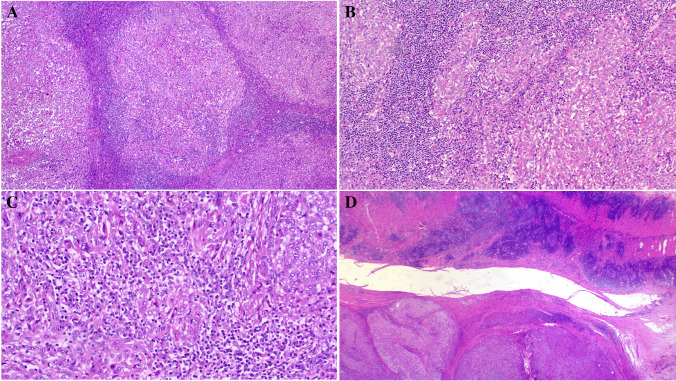
This intra-abdominal tumor showed extensive microlobulation (**A**) and nesting (**B**) associated with prominent mononuclear inflammation (**C**), closely mimicking the recently reported “inflammatory nested sex cord tumors of the testis” [14]. **D** The adjacent colonic muscle wall (upper field) shows an extensive lymphoid reaction. This patient presented with constitutional symptoms and paraneoplastic encephalitis. Note the paracolic location of the tumor in the lower field

**Fig. 6:**
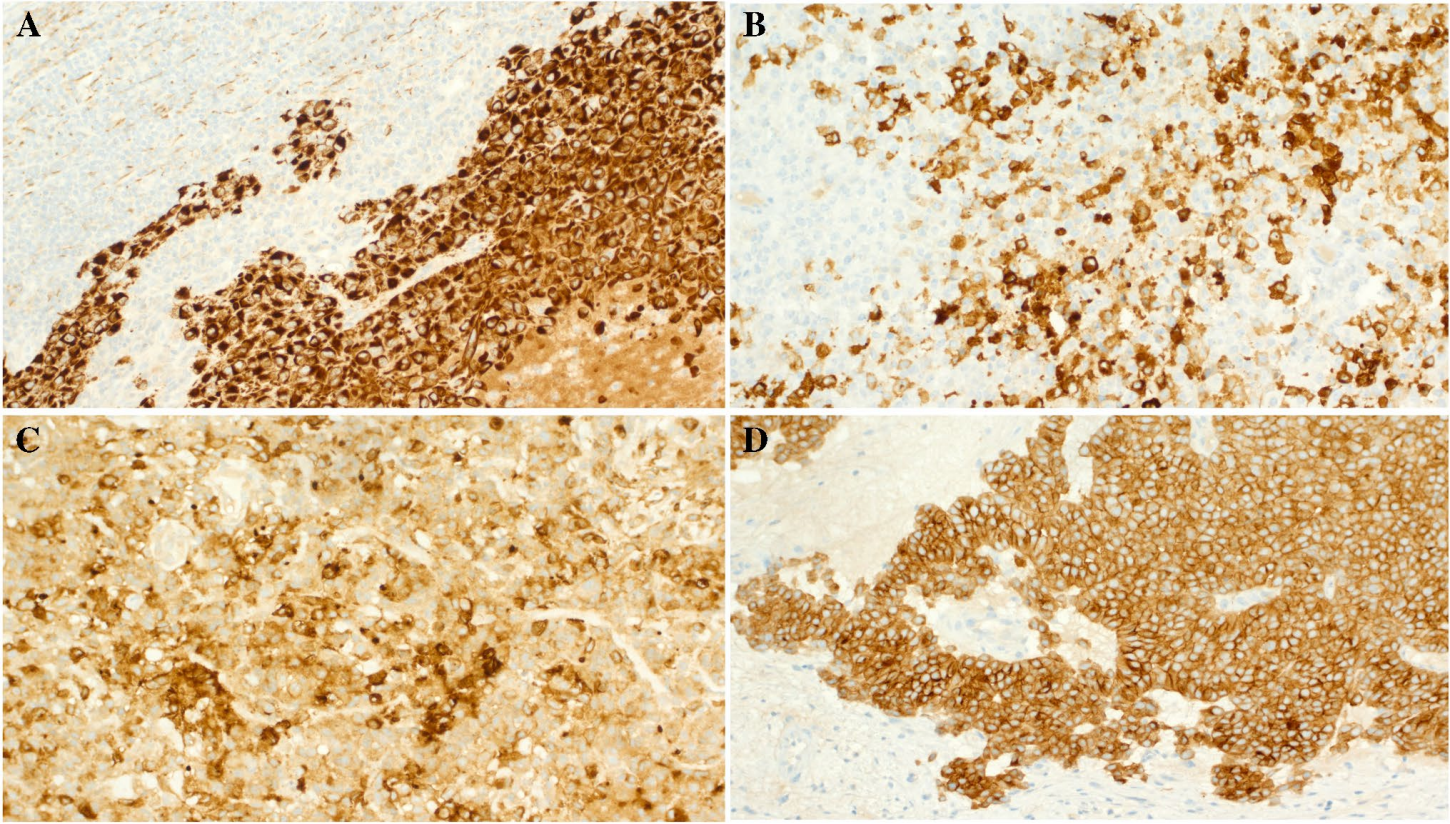
Examples of the immunohistochemical findings in *FET::CREM* fusion neoplasms. **A** Lymph node metastasis from Case 9 (same case as in Fig. 5) showing strong expression of keratin AE1/AE3, note positivity in the lymph node reticulum cells at upper left. **B** EMA was variably expressed in most tumors. **C** Synaptophysin expression was diffuse but variable. **D** Homogeneous ALK expression in one sinonasal tumor

Primary diagnoses were malignant paraganglioma (Case 1), Ewing sarcoma (Case 4), and unclassified neoplasm/ sarcoma in the remainder. The tumor size ranged from 3.5 to 11 cm (median, 6). Histologically, all tumors were not encapsulated and displayed variably infiltrative or irregular borders (except for the kidney tumor (Fig. 1A–D). They revealed comparable morphology, composed of monotonous medium-sized epithelioid or round cells disposed into diffuse sheets, organoid nests, and variably sized and shaped solid aggregates within sparse fibrous stroma. Two tumors showed prominent stromal sclerosis resulting in distinct variably sized compact nests and lobules of tumor cells (Fig. 1A, B). The overall pattern was that of solid or nested monomorphic neoplasms with significant resemblance to solid pattern neuroendocrine tumors. One tumor showed predominantly neuroendocrine-like aggregates and “Zellballen-like nests) that were initially misinterpreted (together with prominent synaptophysin expression) as unusual extra-adrenal paraganglioma (Case 1), resulting in suboptimal initial therapy recommendation (Fig. 2A–D). Foci resembling sclerosing epithelioid fibrosarcoma (SEF) were noted in 8 tumors (Fig. 3A, C). Amorphic collagen deposits occasionally mimicking amianthoid fibers were noted in the stroma of two cases (Fig. 3B). Pseudoalveolar pattern resulting from central discohesion of cells was noted at least focally in all cases (Fig. 3D) and was prominent in one case (Fig. 3A, C). Other patterns that were noted in most cases include highly cellular solid areas with larger clear to pale-eosinophilic epithelioid cells (Fig. 4A) and small round dark stained cells with single-cell file pattern (Fig. 4B). Foci of tumor cell necrosis were seen in all tumors (subtle in some while being extensive in others; Fig. 4C). The mitotic activity ranged from 1 to 8 mitoses in 10 HPFs (median, 4; (Fig. 4D). Notably, all the tumors, but one, lacked myxoid changes. The one renal tumor showed variable microcystic myxoid stromal change (Fig. 3B). Peripheral lymphoid cuffs and aneurysmal and hemorrhagic changes characteristic of AFH were absent in all cases. One intra-abdominal tumor showed prominent intratumoral inflammation composed predominantly of lymphocytes and plasma cells and showed prominent focal nesting indistinguishable from the recently reported “inflammatory nested sex cord tumor of the gonads” [14] (Fig. 5A–C). This tumor showed extensive lymphoid reaction within the adjacent wall of the large intestine (Fig. 5D). This patient presented with constitutional symptoms and paraneoplastic encephalitis. One sinonasal tumor (Case 4) showed variable cystic stromal spaced containing eosinophilic secretory material and a few erythrocytes (Fig. 1D). Of five cases with sampled regional lymph nodes or with a few lymph nodes incidentally found in the resection specimens, two cases (40%) had positive lymph nodes (one extra-abdominal and one abdominal) (Fig. 6A).

### Immunohistochemical findings

The immunohistochemical findings of these tumors were generally nonspecific, but mostly reproducible. Notably, the immunophenotype of the two subcohorts significantly overlapped (Tables 2 and 3). The tumors expressed EMA (7 of 8; Fig. 6B), keratin AE1/AE3 (5 of 9; Fig. 6A), CD99 (4 of 7), MUC4 (2 of 8), synaptophysin (3 of 9; Fig. 6C), ALK (3 of 8; Fig. 6D), chromogranin (1 of 8), CD34 (3 of 6), CD30 (1 of 6), PAX8 (1 of 7), and inhibin (1 of 7). All tested tumors were negative for desmin (0 of 8), S100 (0 of 8), and SOX10 (0 of 8).

**Table 2:** Pathological and immunohistochemical findings in extra-abdominal (1–5) and intra-abdominal (6–9) *FET::CREM* fusion positive unclassified neoplasms

No	Necrosis	Mitoses/10 HPFs	AE1/3	EMA	MUC4	ALK	Desmin	Synaptophysin	Chromogranin	CD30	CD34	Inhibin	PAX8	S100	SOX10	CD99	**Gene fusion**
1	Present	8	-	-	-	+	-	+	-	-	-	-	+ (wk)	-	-	-	EWSR1::CREM
2	Present	2	-	-	-	+	-	-	-	-	+ (F)	-	-	-	-	+	EWSR1::CREM
3	Present	6	+	+	+	+	NA	+	NA	NA	-	NA	NA	NA	-	NA	EWSR1::CREM
4	Present	5	+ (F)	+	-	+	-	+	+ (F)	-		-	+	-	-	+	EWSR1::CREM
5	Present	2	-	+	-	-	-	-	-	-	+ (F)	-	-	-	-	-	EWSR1::CREM
6	Present	1	+ (F)	+	+	-	-	-	-	NA	+	+ (F)	-	-	NA	NA	EWSR1::CREM
7	Present	4	+ (F)	+	-	-	-	-	-	-	-	-	-	-	-	+ (F)	EWSR1::CREM
8	Present	1	-	+	-	-	-	-	-	-	-	-	-	-	-	-	EWSR1::CREM
9	Present	5	+	+	+	-	-	-	-	+	-	-	-	-	-	+	FUS::CREM

*F* focal, *NA* not available

**Table 3:** Compared clinical and immunohistochemical findings in extra-abdominal and intra-abdominal *FET::CREM* fusion neoplasms

Feature/positive marker	Extra-abdominal tumors	Intra-abdominal tumors	All
Age range (median)	31–62 (38)	10–75 (28)	10–75 (34)
Female:male ratio	3:2	4:0	7:2
Follow-up/aggressive course	21–52 mo/1 of 3 DOD	21–52 mo/1 of 2 AWPD	21–52 mo/ 2 of 5 with PD
Keratin AE1/AE3	2/5	3/4	5/9
EMA	3/4	4/4	7/8
MUC4	1/4	2/4	3/8
ALK	3/4	0/4	3/8
Desmin	0/4	0/4	0/8
Synaptophysin	3/5	0/4	3/9
Chromogranin	1/4	0/4	1/8
CD30	0/3	1/3	1/6
CD34	2/2	1/4	3/6
Inhibin	0/3	1/4	1/7
PAX8	1/3	0/4	1/7
S100	0/4	0/4	0/8
SOX10	0/5	0/3	0/8
CD99	2/4	2/3	4/7

*AWPD*, alive with progressive disease; *DOD*, died of disease; *mo*, months; *PD*, progressive disease

### Molecular findings

Targeted RNA sequencing revealed in-frame *FET::CREB* fusions in all cases (Table 4). Specifically, 8 tumors harbored an *EWSR1::CREM* and one tumor a *FUS::CREM* in-frame fusion. The fusion breakpoints involved exon 16 (*n* = 3), exon 14 (*n* = 2), and, in one case, each exon 13, 11, and 7 of *EWSR1*. In the one tumor with *FUS* fusion (Case 9), exon 7 was involved. The breakpoints of *CREM* corresponded to exon 6 (*n* = 4), exon 7 (*n* = 4), and exon 3 (*n* = 1).

**Table 4:** Gene breakpoints and exons involved in extra-abdominal (1–5) and intra-abdominal (6–9) *FET::CREM* fusion neoplasms

No	Breakpoints/exons
1	*EWSR1* (NM_013986.4) exon 14::*CREM* (NM_183011.2) exon 7
2	EWSR1 (NM_005243.3) exon 13::*CREM* (NM_181571.2) exon 7
3	*EWSR1* (NM_013986.4) exon 16::*CREM* (NM_183011.2) exon 6
4	*EWSR1* (NM_013986.4) exon 14::*CREM* (NM_183013.3) exon 6
5	*EWSR1* (NM_013986.4) exon 16::*CREM* (NM_181571.2) exon 7
6	*EWSR1* (NM_013986.4) exon 16::*CREM* (NM_183013.3) exon 6
*7*	*EWSR1 (NM_013986.4) exon 11::CREM (NM_183013.3) exon 6*
*8*	*EWSR1 (NM_005243.3) exon 7::CREM (NM_182717.1) exon 3*
*9*	*FUS (NM004960.4) exon 7::CREM (NM_183011.2) exon 7*

## Discussion

Angiomatoid fibrous histiocytoma (AFH) was first described by Enzinger as an unusual fibrohistiocytic sarcoma in a series of 41 cases in 1979 [20]. This was then followed by two larger series (*n* = 108 and 158 cases) published by Costa and Weiss in 1990 [21] and Fanburg-Smith and Miettinen in 1999 [22]. The median/ mean ages were 13 to 20 years. The original biological attributes of AFH in the original Enzinger series (local recurrence and death from disease in 11 of 24 and 3 of 24 patients with follow-up, respectively [20]) have been revised in these larger subsequent series with extended follow-up and likely more critical review of the defining histological features. Notably, local recurrences developed in 12% and 2.3% of patients; all of whom have received incomplete primary excision and were cured by re-excision [21, 22]. Irregular tumor border, head and neck location, and invasion into deep fascia or muscle were found to correlate with local recurrence and metastases [21, 22]. The only death (1% of all cases) was attributed to presumable lung and cerebral metastases, that have not been verified histologically [21]. These observations justified the removal of the modifier “malignant” from the AFH terminology to avoid overprognostication and inappropriate overtreatment [23].

The current cases and those reported recently on intra-abdominal and extra-abdominal unclassified *FET::CREB* fusion neoplasms are at risk of being misclassified as AFH by the non-critical and those overdependent on genotyping. Moreover, a misconception has emerged that expression of EMA, ALK, and desmin in a *FET::CREB* fusion neoplasm indicates AFH, based on the observation that as many as 50% of AFH cases express these markers [22, 23]. However, these assumptions became more fragile as the wide use of RNA-based NGS technologies has uncovered the limited specificity of these aberrantly expressed immunomarkers. Remarkably, a variety of tyrosine kinases (NTRK, ALK) and aberrant proteins (MUC4) have been reported in AFH without detectable underlying structural genetic correlates [24–26]. Indeed, it seems that these aberrantly expressed markers (although fully nonspecific) are in combination valuable context-dependent surrogates not for AFH, but for a variety of fusion-associated malignancies. An example of this is the frequent coexpression of pankeratin and ALK in *FET::TFCP2* fusion aggressive rhabdomyosarcomas [27] and the frequent expression of EMA and keratins (in addition to occasional ALK, MUC4, etc.) in *FET::CREB* fusion intra-abdominal neoplasms [11–14]. Although a subtype of AFH was shown to display small blue round cells with scant cytoplasm, mimicking undifferentiated round cell sarcomas, these tumors show other features of AFH and behave similarly indolent as conventional AFH [22]. Notably, all tumors with available margins in the large series published by Fanburg-Smith and Miettinen were well-circumscribed. Moreover, all tumors were negative for keratins 8/18 [22].

Finally, the cytomorphology of AFH, as defined by Enzinger and confirmed by others, was that of fibroblast-like and histiocytoid cells, lacking other cell features, an observation that has justified the original “fibrous histiocytoma” terminology proposed by Enzinger [20–22]. On the contrary, the recently described *FET::CREB* fusion neoplasms do not share with AFH more than the genotype and the variable expression of the aforementioned aberrant markers.

The current tumors display variable morphological (monotonous epithelioid cells with variably clear cytoplasm, nested architecture with occasional pseudoalveolar and pseudopapillary patterns) and immunophenotypic (variable reactivity for neuroendocrine markers) overlap with MGNET [7]. While these features might be indistinguishable from the tumors under consideration, especially in limited biopsies, MGNETs are reproducibly defined by their obligate S100/SOX10-positive immunophenotype and they differ from classical CCS of soft tissues by anatomic location and lack of specific melanocytic markers (HMB45, MelanA, tyrosinase) [7]. Finally, all MGNET cases reported by Stockman et al. lacked expression of low molecular weight keratins (AE1/AE3), in contrast to the frequent expression of this marker in > 50% of our current cases.

All these recent observations point to aberrant dysregulated immunophenotypes shared by biologically distinct neoplasms within the same genetic pathway. Accordingly, it is no longer justified to lump any unclassified non-CCS, non-MGNET neoplasm carrying a *FET::CREB* fusion into the category of AFH. These evidently aggressive unclassified neoplasms may share subtle morphological/cytological features with AFH, but they lack the key features of AFH. More importantly, and in contrast to AFH, the tumors we are proposing are almost uniformly deep-seated and most have infiltrative borders, features that were judged as either absent or very rare in AFH. The frequently aggressive course resulting in either death or severe morbidity observed altogether in no less than 30–50% of cases underlines the biological distinctness of these tumors from AFH [11–15] and justifies calling them sarcomas.

The first report of unclassified intra-abdominal and extra-abdominal *EWSR1::CREM* fusion sarcoma was published by Yoshida et al. already in 2019, who included one example each among a series of *CREM* fusion neoplasms spanning different entities from different sites [4]. The authors have considered the two examples as unclassified *EWSR1::CREM* fusion sarcomas. The one case concerned a 15-year-old boy with a 10-cm abdominal cavity mass and multiple liver and peritoneal metastases associated with massive ascites. He died of disease with additional bone metastases (despite chemotherapy with the HD-CAV regimen and pazopanib) 18 months later, despite the relatively bland-looking morphology, lack of necrosis, and low mitotic activity (2/10 high-power fields). His tumor was positive for AE1/AE3, EMA, CD56, CD34, ALK, DOG1 (focal), and synaptophysin and was negative for chromogranin A, WT1 (N-terminus), WT1 (C-terminus), myogenin, desmin, NUT, ETV4, CD31, h-caldesmon, smooth muscle actin, claudin-4, MUC4, S100, SOX10, calretinin, BCOR, SALL4, Pan-TRK, NKX2-2, and TTF-1 [4]. The *EWSR1::CREM* fusion was confirmed by RNA-Seq, FISH, RT-PCR, and Sanger sequencing [4]. The extra-abdominal case affected a 63-year-old woman with a 4.5-cm deep soft tissue mass in the left chest wall. The patient was alive without disease 17 months after surgery. Histology showed uniform small round cells with scant eosinophilic cytoplasm within delicate, fibrillary stroma with frequent perivascular pseudorosettes and up to 5 mitoses/ 10 high-power fields, with the absence of necrosis. The tumor cells expressed CD99, synaptophysin, CD56, MUC4, and EMA (focal), but not AE1/AE3, S100, smooth muscle actin, desmin, CD34, chromogranin A, GFAP, NKX2-2, PAX7, and INSM1. The *EWSR1::CREM* fusion was confirmed by RNA-Seq, FISH, RT-PCR, and Sanger sequencing [4].

We herein describe a unique cohort of soft tissue neoplasms characterized by epithelioid/round cell morphology, unusual immunophenotype, and recurrent *EWSR1/FUS::CREM* fusions, occurring in extra-abdominal deep soft tissue (*n* = 2), head and neck (*n* = 3), and intraabdominal (*n* = 4) sites. All did not fit any defined WHO category and were judged as unclassified despite the detected *EWSR1/FUS::CREM* fusion. These tumors are analogous to the intra-abdominal tumors reported in recent studies and are similar as well to the extra-abdominal tumors harboring *EWSR1::ATF1* fusions reported recently by our group [15] and are also similar to the two above detailed cases with *EWSR1::CREM* fusions reported by Yoshida et al. [4].

Taken together, these studies highlight the existence of aggressive *FET::CREB* fusion neoplasms sharing epithelioid/round cell morphology; unusual nonspecific immunophenotype with frequent expression of keratins; EMA; and diverse other proteins including tyrosine kinases (ALK); transcription factors and cell surface antigens (CD30, MUC4, CD99, PAX8, inhibin, CD34); neuroendocrine markers (synaptophysin, chromogranin); and others. These aberrant markers are shared by a variety of distinct low-grade and aggressive entities driven by *FET::CREB* fusions [11–14], tyrosine kinase fusions [28, 29], *FET::TFCP2* fusions [27], and others, and are not indicative of specific histogenetic line of differentiation.

Based on their structural and functional homology, members of the *FET* and the *CREB* gene families function as mutually exclusive gene fusion partners, resulting in the same clinicopathologic entity. Accordingly, any of the *CREB* members (*CREB1*, *ATF1*, and *CREM*) may represent the fusion partner, although with significantly variable frequencies based on the index entity and the anatomic site with *ATF1* being the most prevalent fusion partner in CCS and MGNET, while *CREB1* is the most frequent fusion partner in AFH. However, even within these entities, the fusion partner seems to vary with the morphological pattern, with the majority of myxoid AFH carrying a *CREM* fusion [4]. Likewise, the category of unclassified epithelioid and round cell sarcoma under consideration is predominantly driven by *CREM* fusions (*CREM* >  >  > *ATF1* >  > *CREB1*), mostly fused to *EWSR1* and less frequently to *FUS*.

With our current series, a total of 66 (37 abdominal, 13 testicular, and 16 extra-abdominal) *FET::CREB* fusion neoplasms have been reported. Overall, 21/32 (66%) intra-abdominal, 11/13 (85%) testicular, and 7/10 (70%) extra-abdominal cases have experienced either local recurrence, nodal metastases, distant metastases, or died of disease. In total, 39 of 53 (74%) patients with follow-up had disease progression indicating an aggressive sarcoma.

In summary, we described five extra-abdominal and four intra-abdominal round cell/ epithelioid mesenchymal neoplasms sharing *EWSR1/FUS::CREM* fusions with frequent expression of aberrant epithelial, neuroendocrine and other markers, but distinct from AFH and not fitting with any currently defined mesenchymal entity. Recognition of this aggressive tumor type and its separation from the diverse morphological, immunophenotypic, and genotypic mimics is mandatory for optimizing the establishment of tailored therapeutic strategies. The significant morbidity and mortality justify calling these tumors aggressive sarcomas to avoid under-prognostication and, hence, insufficient treatment. These rare sarcomas should be distinguished from other intra-abdominal neoplasms that frequently (mesotheliomas) or rarely (subsets of dedifferentiated liposarcomas, gastrointestinal stromal tumors, and others) display an epithelioid morphology and express low molecular weight keratins. This is particularly important due to the significantly different therapeutic approaches to these entities.

## Data Availability

The datasets generated during and/or analyzed during the current study are not publicly available, but are available from the corresponding author on reasonable request.
